# PPAR-gamma regulates PFAS-mediated proinflammatory cytokines in lung epithelial cells 

**DOI:** 10.3389/fphar.2026.1779345

**Published:** 2026-02-03

**Authors:** Sadiya Bi Shaikh, Md Imam Faizan, Khursheed Ul Islam, Virender K. Rehan, Irfan Rahman

**Affiliations:** 1 Department of Environmental Medicine, University of Rochester Medical Center, Rochester, NY, United States; 2 Division of Neonatology, Lundquist Institute for Biomedical Innovation at Harbor-UCLA Medical Center, David Geffen School of Medicine at UCLA, Torrance, CA, United States

**Keywords:** epithelial cells, inflammation, lungs, PFAS, PPARγ

## Abstract

**Background:**

Per and polyfluoroalkyl substances (PFAS), including the legacy compound perfluorooctanesulfonic acid (PFOS), are persistent organic pollutants with long biological half-lives. Emerging evidence suggests a significant accumulation of PFAS/PFOS in the human lung, potentially contributing to inflammation and altered immune responses. However, the role of peroxisome proliferator-activated receptor gamma (PPARγ) signaling in PFAS/PFOS-induced pulmonary toxicity remains unclear.

**Methods:**

Primary human bronchial epithelial (NHBE) cells were exposed to 15 µM binary PFAS mixture (PFOS + PFOA) or quaternary mixture (PFOS, PFOA, PFHxS, GenX) with or without the PPARγ antagonist (15 µM) and/or the PPARγ agonists rosiglitazone (10 µM) or pioglitazone (10 µM) for 24 h. BALB/c mice were orally administered PFOS (2 mg/kg/day) or vehicle control for 2 weeks.

**Results:**

In NHBE cells, PFAS exposure significantly increased IL-6 and IL-8 secretion. Treatment with rosiglitazone or pioglitazone reversed these cytokine increases, whereas co-treatment with the PPARγ antagonist elevated IL-6 and IL-8 levels compared to PFAS exposure alone in epithelial cells. PFOS exposure in mice caused a reduction in lung PPARγ protein levels, while PPARα expression remained unchanged.

**Conclusion:**

These findings demonstrate that PFAS-induced pro-inflammatory cytokines is mediated, at least in part, through PPARγ signaling, and that pharmacological activation of PPARγ signaling can attenuate PFAS-triggered pro-inflammatory cytokine responses in lung epithelial cells.

## Introduction

Per- and polyfluoroalkyl substances (PFAS), the so called “forever chemicals” are ubiquitous in the environment and throughout biological trophic levels, owing to their use in products, such as food-contact materials, non-stick cookware, firefighting foams, stain- and water-resistant fabrics, and personal-care items (Wei et al., 2018; Domingo, 2025; Gl et al., 2020). PFAS are classified by carbon-chain length: long-chain (C > 6), short-chain (4 < C ≤ 6), and ultra-short-chain (C = 2–3), in which nearly all hydrogens are replaced by strongly electronegative fluorine atoms. The resulting C–F bond is the strongest covalent bond in organic chemistry, conferring exceptional chemical and thermal stability. Legacy PFAS, such as perfluorooctanoic acid (PFOA) and perfluorooctanesulfonic acid (PFOS) contain eight carbons (C8), whereas more recently adopted short-chain congeners, including perfluorohexanesulfonic acid (PFHxS), perfluorohexanoic acid (PFHxA), and hexafluoropropylene-oxide dimer acid ammonium salt (HFPO-DA, “Gen X”), contain three to six fully fluorinated carbons (Wei et al., 2018; Domingo, 2025; Gl et al., 2020). PFAS mixtures, including binary combinations like PFOS + PFOA and broader mixtures containing PFOS, PFOA, PFHxS, and PFNA, are commonly found in environmental samples and represent typical exposure scenarios (Post et al., 2024).

Emerging evidence suggests a substantial burden of PFAS in the human lung, which may impact inflammation and allergic responses (Nielsen et al., 2024a; Lucas et al., 2022; Lucas et al., 2024a; Lucas et al., 2024b; Dragon et al., 2023; Kvalem et al., 2020; Luo et al., 2020; Pérez et al., 2013; Qin et al., 2017; Sorli et al., 2019). We have recently shown that developmental PFAS exposure alters airway epithelial permeability and inflammatory-allergic responses in epithelial cells and mouse lungs (Lucas et al., 2022; Lucas et al., 2024a; Lucas et al., 2024b). PPARγ, a nuclear hormone receptor, regulates airway epithelial cell development, epithelial-mesenchymal interactions, and pulmonary inflammatory responses (Pérez et al., 2013; Qin et al., 2017; Sorli et al., 2019; Stark et al., 2021). PPARγ is known to play an important role in lung development and maturation (Reddy et al., 2012; Rehan and Torday, 2012). PFAS may interact with PPARγ. However, the specific role of PPARγ signaling in regulating the inflammatory response to PFAS exposure remains unclear. Here, we show that lung PPARγ levels are reduced after PFAS/PFOS exposure both *in vivo* and *in vitro* models. Furthermore, *in vitro* studies demonstrate that a PPARγ agonist blocks PFAS-induced increase in inflammatory cytokines that were in turn in turn reversed by concurrent treatment with a PPARγ antagonist.

## Materials and methods

### Ethics approval statement: institutional biosafety and animal protocol approval

The University of Rochester’s Institutional Biosafety Committee approved the laboratory protocols under Rahman/102054/09-167/07-186, with the identification code 07-186. The mouse study was reviewed and approved by the University Committee on Animal Research (UCAR) at the University of Rochester, Rochester, NY, under the UCAR protocol 102,204/UCAR-2007-070E.

### Cell culture and treatments

Normal human bronchial epithelial cells (NHBE; Lonza CC-2541) were treated with 15 µM PFOS (Sigma, Cat # 77282), 10 µM rosiglitazone (ROSI) (Sigma, Cat# R2408), 10 µM pioglitazone hydrochloride (PIO) (Sigma, Cat #E6910), or 15 µM PPARγ antagonist III, G3335 (Sigma, Cat# 516566). PFAS treatments were also performed in combination exposures, including a binary mixture of PFOS and PFOA (5 µM each), and a quaternary mixture consisting of PFOS, PFOA, PFHxS (Cayman, CAS 355-46-4), and hexafluoropropylene oxide dimer acid (HFPO-DA or GenX) (Cayman, CAS 13252-13-6). Cells were exposed in a two-step sequence. First, cells were treated with PFOS (15 μM) for 24 h to induce an inflammatory response. After this initial exposure, cells were washed with PBS and then treated with either the PPARγ agonist (ROSI or PIO) or the PPARγ antagonist, and were harvested 48 h after this second treatment. This PFOS and agonist/antagonist sequence was used to assess how modulation of PPARγ influences PFOS-induced cytokine release. Culture supernatants (cell-free media) were collected at the end of the treatment period and used to measure IL-6 and IL-8 levels by ELISA.

### Animals and treatments

The University Committee on Animal Resources at the University of Rochester Medical Center (URMC) approved the study for all animal experiments. Both male and female mice were included in this study (Lucas et al., 2024a; Lucas et al., 2024b). All mice were housed at the University of Rochester Medical Center vivarium facility with a 12-h light/12-h dark cycle. Treatments were performed as previously described (Lucas et al., 2024a; Lucas et al., 2024b).

### Quantification of pro-inflammatory cytokines by ELISA

To assess the release of inflammatory cytokines by the treated and untreated cells, IL-6 (Cat# CHC1263, Invitrogen, Waltham, MA) and IL-8 (Cat# CHC1303, Invitrogen) levels were measured using ELISA according to the manufacturer’s protocol.

### Immunoblotting

Western blot analyses were performed as previously described (Lucas et al., 2024a; Lucas et al., 2024b). Briefly, lung tissue and cell lysates were prepared in RIPA lysis buffer (Sigma, Cat# 20-188). Homogenates were clarified by centrifugation at 13,000 rpm for 30 min at 4 °C, and the supernatants were collected for protein quantification using the BCA assay. Equal amounts of protein were resolved on 10% SDS-PAGE gels and transferred to PVDF membranes (Bio-Rad, Cat# 162-0177) using a wet transfer. Membranes were blocked in blocking buffer (Bio-Rad, Cat# 12010020) for 40 min at room temperature, then incubated overnight at 4 °C with primary antibodies anti-PPARα (mouse; Proteintech, Cat# 66826-1-Ig) and anti-PPARγ (rabbit; CST, Cat# 2435S), each at 1:1000 with gentle agitation. After washing in 1× TBST, membranes were incubated with HRP-conjugated secondary antibodies (Bio-Rad, Cat# 1706515 and 1706516) at 1:10,000, washed, and developed using SuperSignal™ Femto ECL substrate (Thermo Fisher, Ref# 34096).

### Statistical analysis

All experiments involving more than two groups were analyzed using one-way ANOVA. For each analysis, the overall between-group p-value is reported to indicate whether group differences were statistically significant. When pairwise comparisons were made, primarily between the vehicle control and individual treatment groups (PFOS, agonist, or antagonist conditions), we applied Tukey’s Honest Significant Difference (HSD) *post hoc* test. All statistical analyses were performed using GraphPad Prism (version 10), and Data are presented as mean ± SEM. A *p*-value <0.05 was considered statistically significant.

## Results

### PFOS exposure reduces PPARγ abundance in mouse lungs and lung epithelial cells

**FIGURE 1:**
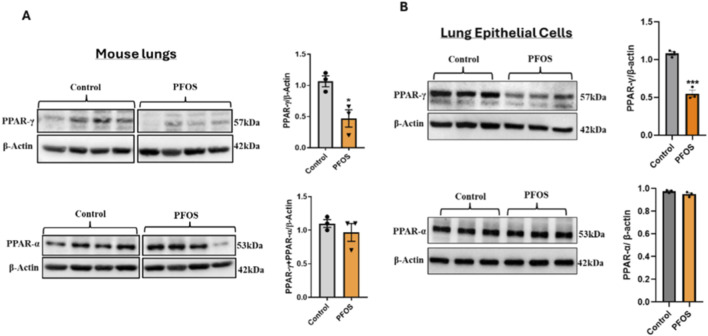
PFOS Exposure Reduces PPARγ Abundance in Mouse Lungs and Lung Epithelial Cells **(A)** Developmental PFOS exposure (2 mg/kg/day) reduces PPARγ protein expression in mouse lung tissue. Bar graphs represent the relative fold change of PPARγ and PPARα protein levels normalized to β-actin. Data are presented as mean ± SEM (n = 3 per group); *p < 0.05 vs. control. **(B)** Representative Western blots of PPARγ and PPARα in primary human bronchial epithelial (NHBE) cells exposed to PFOS (15 µM). Quantification of band intensities normalized to β-actin is shown in bar graphs. Data are expressed as mean ± SEM (n = 3); ***p < 0.001 vs. control.

PFOS exposure led to a significant reduction in PPARγ protein expression, without affecting PPARα levels, in the whole lung tissue of exposed mice as demonstrated by Western blot analysis (Figure 1A). Because PPARγ signaling is a key determinant of lung epithelial cell differentiation and function, and lung epithelial cells are affected by PFOS exposure (Lucas et al., 2022), we next examined the effect of PFOS on PPARγ expression in primary human lung epithelial cells. Consistent with our *in vivo* findings, PFOS exposure significantly reduced PPARγ abundance without altering PPARα levels (Figure 1B). These results indicate that legacy PFOS selectively downregulates PPARγ in both *in vivo* and *in vitro* models, implicating PPARγ signaling as a potential mediator of PFAS-induced pro-inflammatory responses.

### PPARγ agonists suppress PFOS-induced IL-6 and IL-8 secretion by lung epithelial cells

**FIGURE 2:**
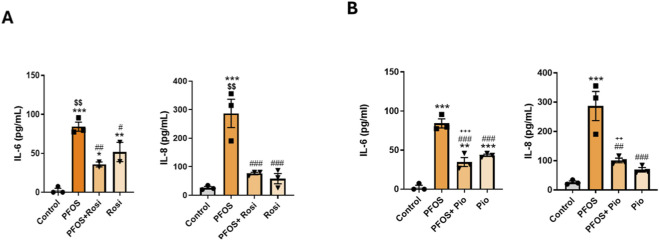
PPARγ Agonists Attenuate PFOS-Induced Inflammatory Cytokines: Primary lung epithelial cells were treated with PFOS (15 µM), rosiglitazone, or pioglitazone HCl (10 µM). **(A)** IL-6 and IL-8 levels measured by ELISA following PFOS and rosiglitazone treatments. **(B)** IL-6 and IL-8 levels measured by ELISA following PFOS and pioglitazone treatments. ***p < 0.001, **p < 0.01, *p < 0.05 vs. control; ^###^p < 0.001, ^##^p < 0.01, ^#^p < 0.05 vs. PFOS; ^$$^p < 0.01 vs. rosiglitazone; ^+++^p < 0.001 vs. pioglitazone.

Human bronchial epithelial cells were exposed to PFOS with or without the PPARγ agonists ROSI or PIO. PFOS exposure markedly increased the secretion of the inflammatory cytokines IL-6 and IL-8 by primary lung epithelial cells, as measured by ELISA. Co-treatment with ROSI significantly reduced PFOS-induced increases in IL-6 and IL-8 levels compared with PFOS alone. Interestingly, ROSI treatment alone produced a modest but insignificant increase in cytokine levels (Figure 2A). Similar effects were observed with PIO, where co-treatment significantly decreased PFOS-induced cytokine secretion, and PIO alone treatment showed the same trend as seen with ROSI (Figure 2B). Overall, both ROSI and PIO attenuated PFOS-induced inflammatory responses, indicating that PPARγ activation mitigates PFOS-triggered cytokine release, highlighting the potential anti-inflammatory role of PPARγ agonists in blocking PFOS-associated lung injury.

### PPARγ antagonism exacerbates PFOS-induced IL-6 and IL-8 secretion in lung epithelial cells

**FIGURE 3:**
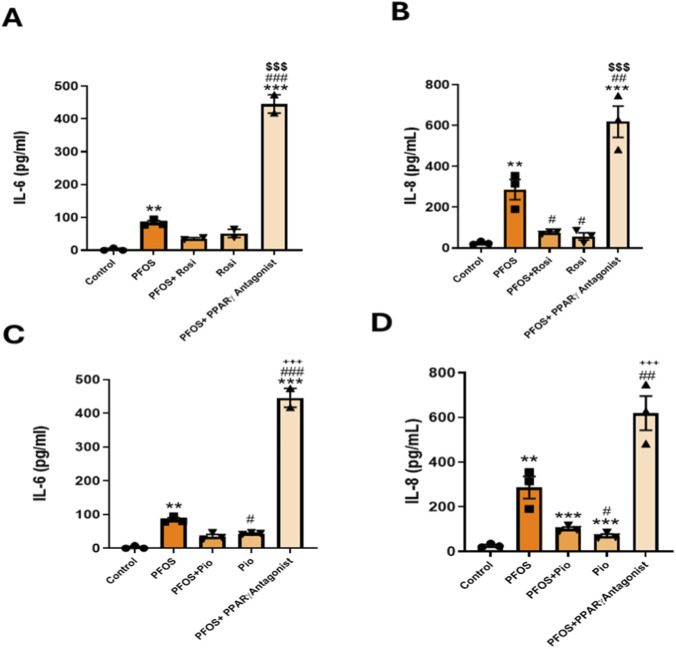
PPARγ Antagonism Exacerbates PFOS-Induced IL-6 and IL-8 Secretion in Lung Epithelial Cells: **(A)** Primary lung epithelial cells were treated with PFOS (15 µM), rosiglitazone, or pioglitazone HCl (10 µM) and PPARγ Antagonist (15 µM). **(A,B)** IL-6 and IL-8 levels measured by ELISA following PFOS and rosiglitazone treatments. **(C,D)** IL-6 and IL-8 levels measured by ELISA following PFOS and pioglitazone treatments. ***p < 0.001, **p < 0.01, *p < 0.05 vs. control; ###p < 0.001, ##p < 0.01, ^#^p < 0.05 vs. PFOS; ^$$$^p < 0.001 vs. rosiglitazone; ^+++^p < 0.001 vs. pioglitazone.

Having observed that PPARγ agonists attenuate PFOS-induced inflammatory responses, we next examined whether blocking PPARγ would exacerbate these responses and negate the protective effects of PPARγ agonists. As above, human bronchial epithelial cells were exposed to PFOS with or without the PPARγ agonists ROSI or PIO, and with or without a PPARγ antagonist (antagonist III, G3335). PFOS exposure significantly increased IL-6 and IL-8 secretion. Co-treatment with ROSI or PIO suppressed these PFOS-induced increases; however, this protective effect was completely abolished by co-treatment with the PPARγ antagonist (Figures 3A–D). suggesting a key role of PPARγ signaling in regulating the inflammatory response of bronchial epithelial cells.

### PPARγ agonist reduces inflammatory cytokine release caused by PFAS mixtures in lung epithelial cells

**FIGURE 4:**
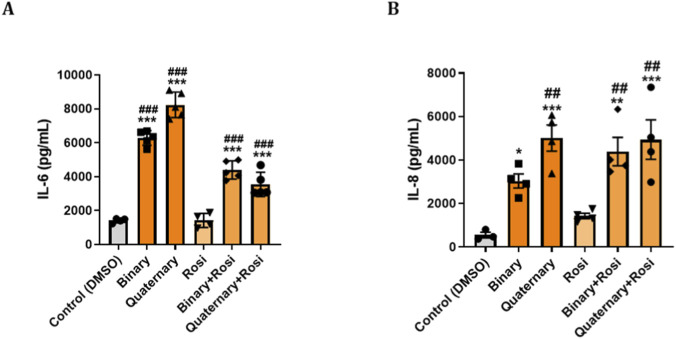
PPARγ Agonist Modulates Cytokine Responses to PFAS in Primary Human Bronchial Epithelial (NHBE): Primary NHBE cells were seeded and treated with various combinations of PFAS and a PPARγ agonist (rosiglitazone). PFAS exposures included a binary mixture (PFOS + PFOA) and a quaternary mixture (PFOS, PFOA, PFHxS, and GenX), designed to mimic environmental co-exposures. Treatments were applied at 0 h and repeated at 24 h. Supernatants were collected at 48 h for ELISA-based quantification of pro-inflammatory cytokines. **(A,B)** Bar graphs show levels of IL-6 and IL-8 under different treatment conditions in response to binary PFAS and quaternary PFAS, and IL-8 secretion under the same respective conditions. Rosiglitazone treatment partially attenuated IL-6 and IL-8 responses, particularly under quaternary exposure condition. Data are presented as mean ± SEM. ***p < 0.001, **p < 0.01, *p < 0.05 vs. control; ^###^p < 0.001, ^##^p < 0.01, vs. Rosi.

Primary human bronchial epithelial cells were exposed to binary (PFOS + PFOA) and quaternary (PFOS, PFOA, PFHxS, GenX) PFAS mixtures, both of which significantly increased IL-6 and IL-8 secretion compared with controls. As shown in Figure 4A, both mixtures markedly elevated IL-6 production, with the quaternary mixture inducing the highest levels. Co-treatment with the PPARγ agonist ROSI significantly reduced IL-6 secretion under both exposure conditions, although levels remained above control values. A similar pattern was observed for IL-8 (Figure 4B), where the quaternary mixture again elicited a slightly higher response than the binary mixture. ROSI co-treatment though attenuated IL-6 levels but had no effect on IL-8 release.

## Discussion

PFAS, including PFOS, have been widely detected in human serum, and several reports indicate their accumulation in lung tissue (Pérez et al., 2013). Epidemiologic studies have linked PFAS exposure to heightened asthma risk (Calafat et al., 2007; Jackson-Browne et al., 2020; Zeng et al., 2019; Dong et al., 2013), with serum PFAS concentrations correlating with asthma incidence (Zeng et al., 2019; Dong et al., 2013). In rodent models, PFAS exposure elevates Th2 cytokines, augments eosinophilic inflammation (Yang et al., 2021; Dong et al., 2011), and amplifies ovalbumin-induced allergic airway inflammation (Yang et al., 2021; Lee et al., 2018). PFAS exposure disrupts epithelial and immune homeostasis through both direct and systemic mechanisms. Recently, we demonstrated the sex-dependent effects of developmental PFOS exposure on airway allergic responses in mice (Lucas et al., 2024a; Lucas et al., 2024b). Together, these observations underscore the clinical relevance of elucidating PFAS-driven mechanisms underlying aberrant lung development and immune modulation (Gl et al., 2020; Sevelsted et al., 2023; Rafiee et al., 2024; Solan and Park, 2024; Shi et al., 2023; Chen et al., 2024; Kornher et al., 2025). However, the exact mechanisms by which PFAS induce pulmonary toxicity, particularly their impact on PPARγ signaling, a key pathway involved in lung development and inflammation regulation, remain poorly understood.

We found that PFOS exposure (a legacy PFAS) decreases PPARγ protein levels in mouse lungs, while there was no change in the abundance of PPARα protein levels between treated and control groups. To investigate whether PPARγ regulates PFOS-induced epithelial inflammation, we modulated this pathway in lung epithelial cells.

PPARγ agonists (ROSI, PIO) prevented the PFOS-driven increases in IL-6 and IL-8, whereas co-treatment with a PPARγ antagonist further exacerbated these cytokine levels relative to PFOS alone. Together, these data identify PPARγ signaling as a central attenuator of PFOS-induced inflammatory responses in lung epithelium. We then examined the effects of mixture of environmentally relevant, medium-to long-chain PFAS commonly found in the environment, including PFOA, PFHxS, PFOS, and HFPO-DA GenX, an industrial replacement for the legacy PFAS- PFOA. We studied the effects of a mixture of medium to long-chain PFAS found in the environment, i.e., PFOA, PFHxS, PFOS, and HFPO-DA(Gen-X, an industrial replacement for the straight-chain PFAS and PFOA). Both binary and quaternary PFAS mixtures robustly induced pro-inflammatory cytokine release by lung epithelial cells, and that PPARγ agonists partially mitigated this effect, particularly for IL-6, highlighting the role for PPARγ signaling in modulating PFAS-induced inflammation. These results are consistent with prior reports of PFOS-mediated lung inflammation (Lucas et al., 2022; Lucas et al., 2024a; Lucas et al., 2024b) and the anti-inflammatory actions of ROSI and related analogs (Kang et al., 2011; Ward et al., 2006; Rehan et al., 2006; Sakurai et al., 2018; Lee et al., 2020; Anderson et al., 2016). IL-6 and IL-8 are regulated through distinct pathways. IL-6 primarily via STAT3 and IL-8 through NF-κB and PPARγ agonists differentially modulate these signals (Ricote et al., 1998; Ryoo et al., 2004; Cronin et al., 2016). This explains why ROSI/PIO produce stronger suppression of IL-8 (NF-κB–dependent), but may have a more variable effect on IL-6 due to PPARγ-STAT3 interactions (Cronin et al., 2016). Given the evolving PFAS landscape, our findings underscore the need to examine the effects of ultrashort-chain PFAS, which are increasingly replacing legacy PFAS (PFOS) in the real world, on lung immune-inflammatory responses (Cheng et al., 2026), especially in the developing lung (e.g., during various stages of lung development), as well as from mother to child.

This study has several limitations., e.g., all experiments were performed in primary NHBE cells, which model proximal airway responses but do not represent the full heterogeneity of lung cell populations, such as structural alveolar epithelial cells, fibroblasts, and immune cells (involving various T- and B-subsets). It examines PFOS exposure in combination with PPARγ agonists and antagonists in a controlled *in vitro* system, and does not account for chronic or mixed PFAS exposures common in environmental settings. An PPARγ antagonist-only exposure condition was not included in our experimental design; therefore, the independent effects of PPARγ inhibition without PFOS exposure could not be segregated. A single PFOS concentration and exposure sequence were used, limiting broader dose-response and temporal interpretations. Furthermore, it may also be important to assess PPARγ responses independent of receptor ligand binding. Finally, while we focused on PPARγ protein and cytokine outputs, other relevant mechanisms, including transcriptional changes and cross-talk with additional PPAR isoforms, were not examined. Future studies will expand to *in vivo* models including PPARγ deficient mouse, multiple lung cell types, and the inclusion of all pharmacologic controls (as well as the use of PPARγ/PPAR-α mutant cells) to more comprehensively define PFAS-mediated airway toxicity including their deposition/toxicity in other organs (kidney, liver, and spleen), which have now been well documented (Nielsen et al., 2024b; Ryu et al., 2024). Nevertheless, our findings show that PFAS-mediated pro-inflammatory cytokines is mediated, at least in part, through PPARγ signaling in lung epithelial cells.

## Data Availability

The original contributions presented in the study are included in the article/Supplementary Material, further inquiries can be directed to the corresponding author.
