# Supplementary material for: PPAR-gamma regulates PFAS-mediated proinflammatory cytokines in lung epithelial cells
Source: Front Pharmacol. 2026 Feb 3;17:1779345. doi: 10.3389/fphar.2026.1779345 (PMC12910160; doi:10.3389/fphar.2026.1779345)

Figure 1 **A**

Uncropped full blots

PPAR-γ
Top
Upper Panels

PPAR- γ

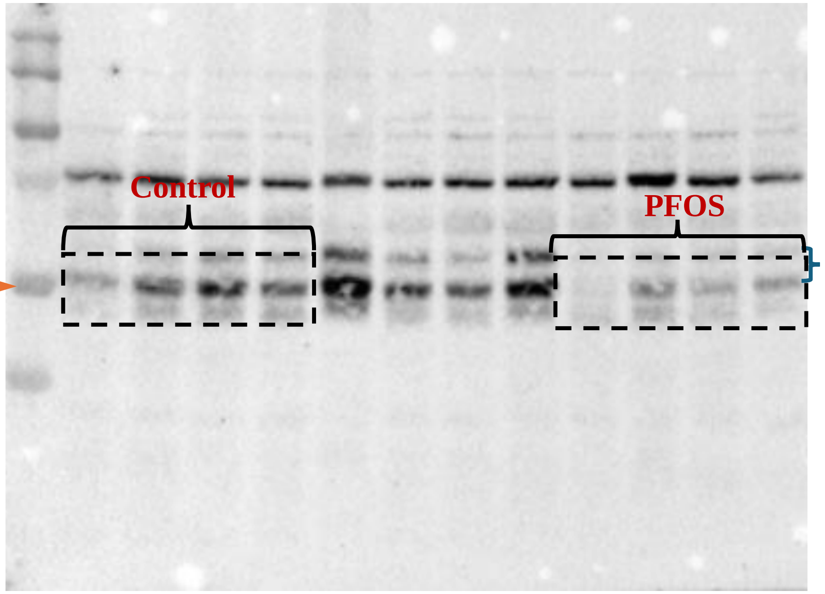

PPAR-α
Bottom:
Upper Panels

PPAR- α

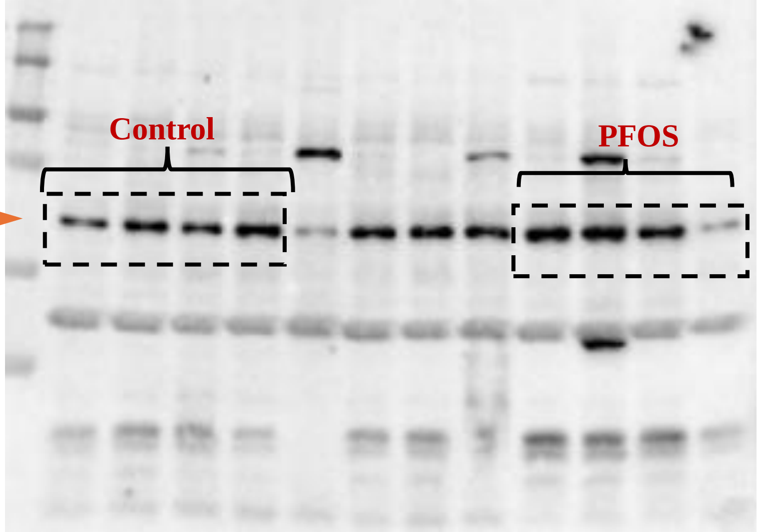

PPAR-γ
Top:
Lower Panels:

β-Actin

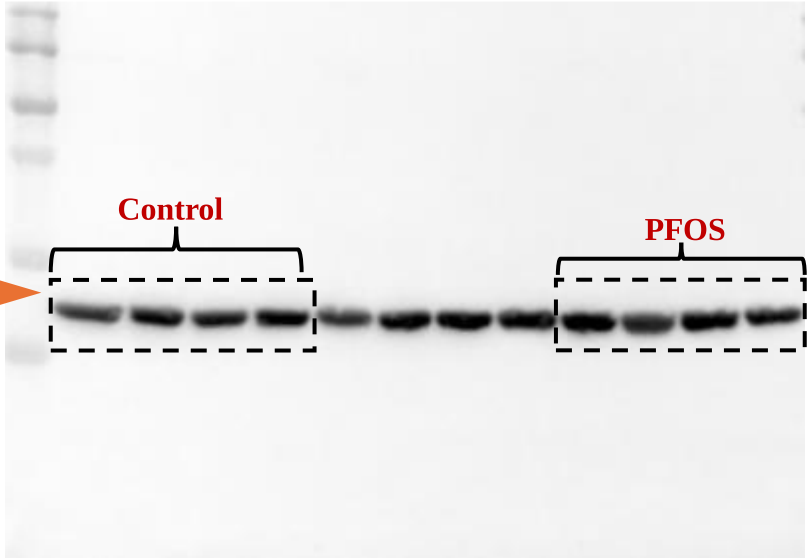

PPAR-α
Bottom:
Upper Panels

β-Actin

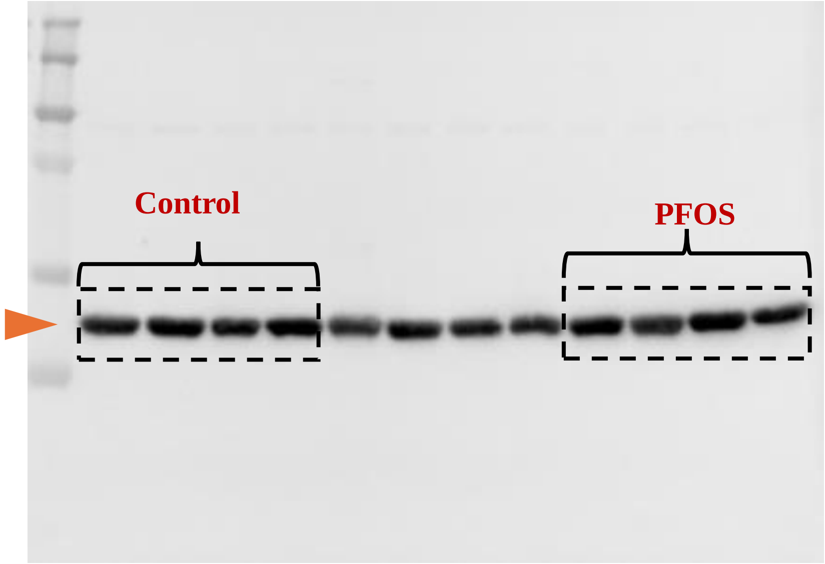

Figure 1 **Uncropped full blots**

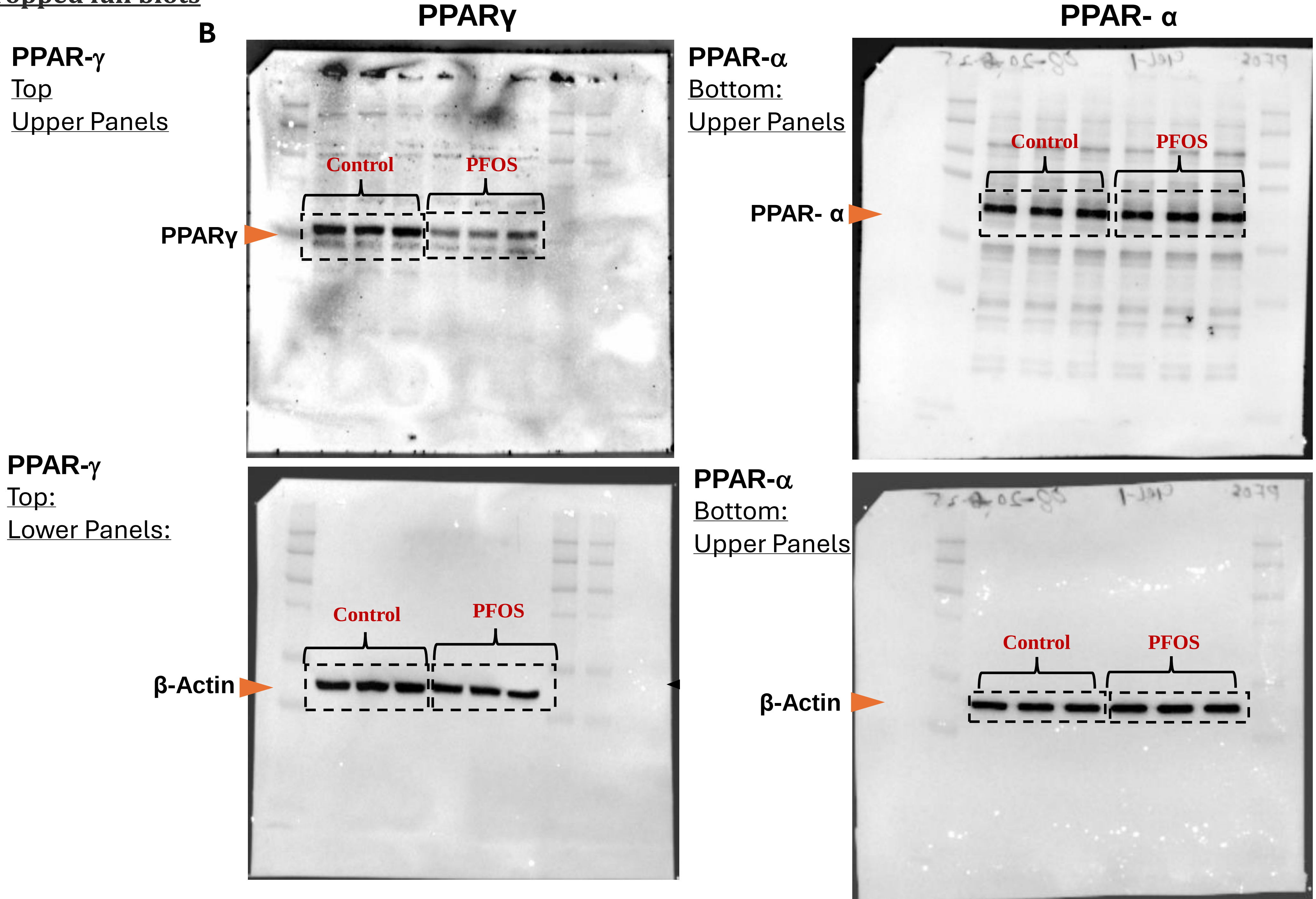

Supplement: Supplementary file 1 [file DataSheet1.pdf]
